# Patterns of gut microbiome composition, function and dynamics in toddlers, adolescents and adults over a three-year period

**DOI:** 10.3389/fmicb.2026.1768977

**Published:** 2026-03-20

**Authors:** Alejandra Rey-Mariño, Susana Ruiz-Ruiz, Nuria Jiménez-Hernández, Xavier Pons, Alejandro Artacho, Pilar Codoñer-Franch, M. Pilar Francino

**Affiliations:** 1 Area de Genòmica i Salut, Fundació per al Foment de la Investigació Sanitària i Biomèdica de la Comunitat Valenciana (FISABIO), València, Spain; 2 Department of Pediatrics, Obstetrics and Gynecology, University of Valencia, València, Spain; 3 Department of Pediatrics, University Hospital Dr Peset, Fundació per al Foment de la Investigació Sanitària i Biomèdica de la Comunitat Valenciana (FISABIO), València, Spain; 4 CIBER en Epidemiología y Salud Pública (CIBERESP), Madrid, Spain

**Keywords:** 16S rRNA gene sequencing, childhood and adolescence, functional profiling, gut microbiome, longitudinal analysis, metagenomics, microbiota dynamics, microbiota stability

## Abstract

Despite their relevance, studies of the long-term stability of the gut microbiome are rare due to the difficulty in following the same individual through long periods of time, particularly during childhood and adolescence. Here, we have been able to analyze microbiome stability throughout a 3-year period in toddlers, adolescents, and adults of the same population, at the levels of taxonomic composition and functional profile. Our analyses show that stability is lower at taxonomical than at functional level in all three age groups, indicating the existence of functional redundancy through time. Considering the entire period of sampling, toddlers were significantly more unstable than the other two groups at the level of taxonomic composition. However, local analyses revealed that low stability for both composition and function was restricted to the time period between 20 and 24 months of age, whereas after this point stability levels in toddlers were similar to those of adolescents and adults. Although the microbiome stabilized at around two years of age in terms of large-scale, rapid changes in diversity, composition, and functional profile, further changes did occur both before and after adolescence. Therefore, adolescence remains a transitional period, in which the abundances of some taxa and functions still differ from adult levels. These include, among others, *Bifidobacterium*, *Streptococcus*, *Bacteroides fragilis* and several members of the Lachnospiraceae, as well as various functions related to energy metabolism. Overall, our results pinpoint the two-years mark as a point of significant stabilization for the gut microbiome, without precluding the further occurrence of important changes in the relative abundance of specific taxa and gene functions both before and after adolescence.

## 1 Introduction

The human gut microbiota is a complex microbial community composed of bacteria, archaea, viruses, and unicellular eukaryotes. These microorganisms encode a large number of genes (over 3 million) and produce thousands of metabolites, contributing essential functionalities to human physiology such as food digestion, protection against pathogens and host immunity regulation (Sekirov et al., 2010; Francino, 2017; Valdes et al., 2018). The structure of the human gut microbiota has been extensively studied in healthy adults and infants, as well as in numerous disease conditions, and it has been proposed that its adult composition is established around 1–4 years of age (Ellis-Pegler et al., 1975; Yatsunenko et al., 2012). However, more recent research has indicated that the gut microbiota is still developing at older ages (Hollister et al., 2015; Cheng et al., 2016; Roswall et al., 2021), with some studies suggesting that microbiota development could continue up to and during adolescence (Agans et al., 2011; Kim et al., 2018; Korpela et al., 2021). Although the study of gut microbiota development has emphasized the analysis of this process in infants, it could indeed be expected that the important anatomical, physiological, and behavioral changes that still occur throughout childhood and adolescence have important effects on the composition and function of this microbial community. Therefore, it is important to establish at what age the microbiota reaches typical adult characteristics in terms of composition, functionality, diversity, and temporal stability. However, few analyses have been carried out in adolescents and most are associated with health disorders (Ferrer et al., 2013; Stanislawski et al., 2018; Mokhtari et al., 2023). In addition, these studies have been based on the relative taxonomic composition of the microbiota, so they lack information on gene composition and functional capacity of the gut microbiome.

In adults, the gut microbiota displays a considerable temporal stability in composition and function (Faith et al., 2013) and this stability has been shown to be important for maintaining health (Lozupone et al., 2012; Martí et al., 2017). However, the question of how temporal dynamics vary at different ages has not been addressed, particularly during life periods in which the microbiota has not yet reached its adult conformation. Understanding these microbiome patterns through childhood and adolescence should help spot alterations that may relate to the early development of metabolic and immune diseases. Here, we used a longitudinal cohort of 60 healthy Spanish volunteers (12 toddlers, 13 adolescents and 35 adults) to study and compare gut microbiota dynamics in these three life stages over a three-year period, at both compositional and functional levels. Toddlers had undergone a long period of breastfeeding, most of them being weaned during the course of the study. On the other hand, most females in the adolescents’ group underwent menarche during this period. This allowed us to explore the contribution of these two major developmental events to the convergence of the microbiota towards adult-like properties.

## 2 Methods

### 2.1 Human subjects and sample collection

We recruited 60 healthy participants belonging to 24 families from Valencia (Spain), including three age groups: adults (W = woman; M = man), adolescents (AA = female; AO = male) and toddlers (NA = female; NO = male). Separate versions of informed consent sheets, adapted to each age group, were read and signed by all adults and adolescents participating in the study.

At the start of the study, average ages for adults (*n* = 35), adolescents (*n* = 13) and toddlers (*n* = 12) were 41.6 years, 11.9 years, and 20 months, respectively (Supplementary Table S1). Adults were recruited among the parents of toddlers and adolescents. None of the toddlers or adolescents were siblings, except participants NA-6 and NO-6, who were twins. Exclusion criteria included suffering gastrointestinal or metabolic diseases and having taken antibiotics within the 3 months before the start of the study. Fecal samples were collected every three to 4 months during 3 years, with a total of 10 sampling time points (see Supplementary Table S1 for sample metadata). All samples were collected by the participants (or by the parents in the case of toddlers); stools were collected in aluminum-foil-covered bidets, potties or diapers and approximately 10 g of material was transferred into sterile 50 mL Falcon tubes (CLS352070) containing 10 mL of RNAlater solution (Invitrogen AM7021). Tubes were stored at room temperature and later at −80 °C in the laboratory until processing. Participants were asked to fill questionnaires with information related to diet, general health, intake of antibiotics, pre- and probiotics, breastfeeding, menarche, lifestyle, etc.

### 2.2 DNA extraction

Fecal samples were defrosted and resuspended in 10 mL of phosphate-buffered saline (PBS [pH 7.2]) (containing, per liter, 8 g of NaCl, 0.2 g of KCl, 1.44 g of Na_2_HPO_4_, and 0.24 g of KH_2_PO_4_, all from Sigma-Aldrich) and then centrifuged first at 2,000 rpm at 4 °C for 5 min to remove fecal debris and then at 13,000 rpm for 2 min in an Eppendorf 5810R centrifuge to pellet bacterial cells. Total genomic DNA from bacteria was extracted using the MagNA Pure LC DNA isolation kit III for Bacteria and Fungi (Cat. No. 03264785001) using the MagNA Pure LC instrument (Roche) according to the instructions of the manufacturer, with addition of a digestion step at the beginning of the protocol using lysozyme (AppliChem A4972) for 30 min at 37 °C followed by cell lysing in 1 mm Zirconium bead tubes (Merck BMSD113210TP) with 500 microliters of phenol:chloroform:isoamyl alcohol (25:24:1) (AppliChem).

### 2.3 16S rRNA gene sequencing, read processing and annotation

The V3-V4 region of the 16S rRNA gene was amplified with forward primer 5′-TCGT CGGC AGCG TCAG ATGT GTAT AAGA GACA GCCT ACGG GNGG CWGC AG-3′ and reverse primer 5′-GTCT CGTG GGCT CGGA GATG TGTA TAAG AGAC AGGA CTAC HVGG GTAT CTAA TCC-3′, with adapter sequences for compatibility with the Illumina Nextera XT Index kit. Amplicons were sequenced with 2×300 bp paired-end reads in a MiSeq System (Illumina) with library preparation following the manufacturer’s guidelines (~120 samples per run yielding ~122,000 reads per sample on average; sequencing statistics are available in Supplementary Table S2). Negative controls were performed at each step of the protocols. Processing, assembly, and annotation of reads followed the workflow of the DADA2 package (v1.8.0) (Callahan et al., 2016) in R (v3.6.0). Raw sequence data were filtered to remove low quality reads and trimmed using the following parameters: maxN = 0, maxEE = c(5,5), truncQ = 0, rm.phix = TRUE and truncLen = c(265,225). A sample inference algorithm was applied to the filtered and trimmed data, and the forward and reverse reads were merged with a minimum of 15 overlapping bases. An amplicon sequence variant table (ASV) was then constructed. Chimeras and human sequences were removed, the latter through the alignment against the human genome (GRCh38.p13) using bowtie2 (v2.4.2) (Langmead and Salzberg, 2012). Taxonomy was assigned by comparison against the SILVA Reference database (release 138) (Quast et al., 2013) in the DADA2 framework, with species-level assignment at 100% similarity or assignment at the deepest possible taxonomic level. BLAST v2.10.1+ (Camacho et al., 2009) was then employed to provide species-level assignations to ASVs that remained unassigned under this strict criterion, using a 97% similarity cutoff. Finally, a contingency table with the ASVs annotated at each taxonomy level and their absolute abundances per sample was obtained (Supplementary Table S2A).

### 2.4 Metagenomic shotgun sequencing, read processing and annotation

Shotgun metagenomic sequencing was performed in the MiSeq System (Illumina) with library preparation following the manufacturer’s guidelines using the 2×300 bp paired-end read protocol (~60 samples per run yielding ~437,000 reads per sample on average; sequencing statistics are available in Supplementary Table S3). Negative controls were performed at each step of the protocols. Preprocessing of the reads, filtering, assembly, and annotation were done using an in-house pipeline that includes publicly available tools. Adapter sequences were removed with cutadapt (v2.9) (Martin, 2011). Prinseq (v0.20.4) (Schmieder and Edwards, 2011) was used to filter and trim the end of the reads by quality. The joining of the overlapping pairs was done with FLASH (v1.2.11) (Magoč and Salzberg, 2011) with a minimum overlap region of 12 nucleotides, and a maximum average value of mismatches of 0.1. Host and ribosomal RNA sequences were removed using bowtie2 (v2.3.5.1) against the human genome and the SILVA Reference database (release 138) respectively. Bacterial reads were then assembled into contigs using megahit (v1.2.9) (Li et al., 2015) and then reads were mapped against the contigs, with a minimum percentage of identity of 95% and a minimum coverage of 80%. Coding regions inside contigs were predicted with Prodigal (v2.6.3) (Hyatt et al., 2010) in the metagenomic mode. For the functional annotation of the predicted ORFs, HMMER (v3.3) (Finn et al., 2011) was used to map these ORFs against the protein models in the TIGRFAM database (v15) (Haft et al., 2001), setting the significance threshold or E-value at 0.001 for hit matches. A contingency table of TIGRFAM annotations and their absolute abundances per sample was generated. Taxonomical annotation was performed by mapping the reads against the NCBI non-redundant protein database using Kaiju (v1.7.3) (Menzel et al., 2016). Furthermore, we developed an in-house pipeline that generates tables relating each read to both its taxonomic assignment and the functional annotation of the ORF to which it maps. To quantify those taxa that contribute genetically to the protein functions encoded in the metagenome, we computed for each ORF detected in a sample the percentage of the mapping reads contributed by different taxa.

### 2.5 Comparison of microbiota diversity, taxonomic composition, and functional profile

Compositional and functional profiles were summarized by age group by plotting the average relative abundances of genera or of TIGRFAM subroles per time point. The vegan R library (v2.5.6) (Oksanen et al., 2019) was used to compute alpha diversity [Shannon index (Shannon, 1948) and Chao1 estimator (Chao, 1984)] at ASV level. We applied the Mann–Whitney *U* test to check for significant differences (*p*-value < 0.05) for alpha diversity estimators, both between different time points within each age group and between age groups at each time point.

Canonical Correspondence Analysis (CCA) and Permutational Multivariate Analysis of Variance (PERMANOVA) were performed on taxonomic and functional data. The Adonis function included in the vegan library was employed to perform PERMANOVA with 600 permutations. For these analyses, aggregated data corresponding to median relative feature abundances (ASVs or TIGRFAMs) for all time points of an individual were used. In the case of CCA and PERMANOVA analyses of potential differences associated to weaning or menarche, we used aggregated data corresponding to median relative abundances of ASVs or TIGRFAMs for all time points of an individual before or after the event. Additionally, Principal Coordinates Analysis (PCoA) was performed on the Jaccard index, Bray-Curtis and Unifrac (weighted and unweighted) distances based on ASV relative abundances from time series data, incorporating time as the third axis in the plot (in addition to principal coordinate 1 and principal coordinate 2) to explore how samples change over time.

### 2.6 Identification of discriminant features among groups

#### 2.6.1 Linear discriminant analysis (LDA)

To identify discriminant features for different groups we first applied the LDA effect size (LEfSe) algorithm (Segata et al., 2011) on genus, species and subrole aggregated data. The algorithm combines Kruskal–Wallis and pairwise Wilcoxon rank-sum tests for statistical significance and feature selection, and we used default parameters for significance (*p*-value < 0.05) and LDA threshold (>2.0).

#### 2.6.2 Sparse partial least squares discriminant analysis (sPLS-DA)

We also employed the sPLS-DA method, implemented in the R package mixOmics (v6.6.2) (Rohart et al., 2017), on aggregated ASV, genus, TIGRFAM and subrole data with the purpose of identifying finer subsets of discriminant features in the data set. Performance and parameter tuning were carried out using 5-fold cross validation repeated 10 and 100 times, respectively. In this last step we set a maximum of ncomp = 6 for metagenome data and ncomp = 2 for amplicon data, as suggested from the performance assessment. The maximal distance was specified for each dataset to predict class membership across all cross-validation runs. The final sPLS-DA models were run after a centered log-ratio transformation to deal with compositional data. To avoid divisions by zero in this step, a *pseudocount* matrix was calculated by adding the smallest non-zero value to the normalized and aggregated count matrix. Clustered Image Maps (CIM) and relevance networks were constructed to visualize the results of the analysis. In CIMs a hierarchical clustering operates simultaneously on the rows and columns of the matrix, based on Euclidean distance between subsets of variables. Relevance networks represent the correlation structure between variables. Networks were saved in a gml format (Graph Markup Language) using the igraph R package (v1.2.4.1) (Csardi and Nepusz, 2006) and input to Cytoscape (v3.8.2) (Shannon et al., 2003) for representation.

#### 2.6.3 Integrative ‘omics analysis

To identify signatures composed of highly correlated features across metagenomic and 16S rRNA gene amplicon datasets, we employed the DIABLO framework (*D*ata *I*ntegration *A*nalysis for *B*iomarker discovery using *L*atent c*O*mponents; Singh et al., 2016) using the ASV and TIGRFAM aggregated *pseudocount* matrices as input. We first fit a DIABLO model without variable selection to assess global performance and choose the number of components for the final DIABLO model. This step was run with 10-fold cross validation repeated 100 times. Relevance networks and Clustered Image Maps were constructed to visualize the features selected by the final model. In addition, a *Circos* plot was constructed to display strong positive or negative correlations (*r* > 0.7) between ‘omic features.

### 2.7 Time series analysis

From the time series data, we wanted to quantify how the microbiota changes over time in each of the age groups, in order to make comparisons within and between groups. To this aim, we employed a variety of strategies described below.

#### 2.7.1 Analyses of diversity and taxonomic composition dynamics with q2-longitudinal

We used the 16S rRNA gene amplicon data and the plugin q2-longitudinal (Bokulich et al., 2018) in the QIIME 2 platform (to analyze alpha diversity dynamics in our longitudinal samples) (Bolyen et al. 2019). We evaluated the difference in alpha diversity values (Shannon index) between the first time point (T1) and the last one (T10) within the same group (Wilcoxon-signed rank test), and tested whether these paired T1-T10 differences were significantly different between age groups (Mann–Whitney *U* test). Linear mixed-effects (LME) models were also used to evaluate the changes in Shannon index over time and across age groups. Finally, machine learning regression (random forests) was employed to identify taxa that were predictive of the different time points in each age group. The longitudinal abundance of each feature was plotted using volatility plots.

#### 2.7.2 Analyses of microbiota stability

In order to study the overall stability in composition and function of the microbiota within toddlers, adolescents and adults, we applied the software *complexCruncher* (Martí et al., 2017). This method is based on the fact that the dynamics of the microbiota fit a Taylor’s power law, meaning that there is a clear correspondence between the mean and dispersion of the relative abundances of taxonomic groups. The parameters of Taylor’s law are the variability of taxa (*V*), which is a direct estimator of fluctuations over time, and the scale index of the power law (*β*), which relates the mean and the dispersion of feature abundances, providing information on the statistical properties of the ecosystem. Both parameters correlate with the stability of the microbiota. *V* represents the maximum variability attainable by a hypothetically dominant taxa (with relative abundance close to 1). If *V* is small, the microbial community is stable; if it is large, the microbiota might be unstable or resilient. Further, if the scaling index β is 1/2, the system behaves like a Poisson distribution, and if it is 1, the system behaves like an exponential distribution. *complexCruncher* calculates these parameters both at compositional (ASVs) and functional (TIGRFAM subroles) levels and constructs the Taylor’s law parameter space, with the variability of the features (*V*) in the x-axis and the scale index (*β*) in the y-axis, both in standard deviation units. *ComplexCruncher* also calculates the rank stability index (RSI), a way to quantify the stability of each of the features (ASVs and subroles) over time in each age group. The RSI is calculated, per feature, as 1 minus the quotient of the number of true rank hops taken divided by the number of maximum possible rank hops, all to the *p* power:


$$\mathrm{RSI}={\left(1-\frac{D}{\left(N-1)(t-1\right)}\right)}^{p}$$

where *D* is the total number of rank hops taken by the feature, *N* is the number of features that have been ranked, and *t* is the number of time samples. RSI is strictly 1 for a feature whose range never changes over time and strictly 0 for an element whose range oscillates between the extremes. We performed CCA and PERMANOVA based on RSIs to check if we could separate the microbial communities of the different age groups based on the distribution of stability values among features.

Finally, we applied another strategy to study whether microbiota stability within the different age groups changed throughout the sampling time span. To this aim, we calculated the Jaccard index for all pairs of consecutive samples from the same subject, both at ASV and TIGRFAM levels. The Jaccard index is a beta-diversity metric that indicates the similarity between two samples or data sets by computing the fraction of shared features between them, and it ranges from 0 to 1; the closer to 1, the more similar the two samples. We performed Mann–Whitney *U* tests both between and within age groups to test the significance of the differences in the Jaccard index (1) between comparisons of consecutive time points within the same age group (within-group comparison) and (2) between groups for the same consecutive time points comparison (between-groups comparison).

## 3 Results

### 3.1 Gut microbiota 16S rRNA gene and metagenomic sequencing in toddlers, adolescents, and adults

Stool samples were collected from each participant every 3–4 months during a period of 3 years. Compliance with the sample collection schedule was high, so that we obtained a total of 496 samples over 10 timepoints (119 samples from toddlers, 101 samples from adolescents and 276 samples from adults). We obtained a total of 60,687,163 reads for 16S rRNA gene sequencing (mean of 124,063 reads per sample in toddlers, 122,020 reads per sample in adolescents and 117,555 reads per sample in adults) and a total of 216,779,268 reads for shotgun metagenomics (mean of 485,157 reads per sample in toddlers, 411,977 reads per sample in adolescents and 425,492 reads per sample in adults) (Supplementary Tables S2B-S3). To avoid confounding factors, samples from women who became pregnant during sampling time (subjects W-7, W-8, W-11, and W-12) were not included in subsequent analyses. On the other hand, samples from women who were breast-feeding during part of the sampling period (W-1, W-2, W-3, W-9, and W-10) were incorporated into the analyses; the taxonomic and functional composition of the microbiome for these women did not deviate from that of other adults in the data set.

### 3.2 Global composition and diversity of the gut microbiota at different ages

**Figure 1:**
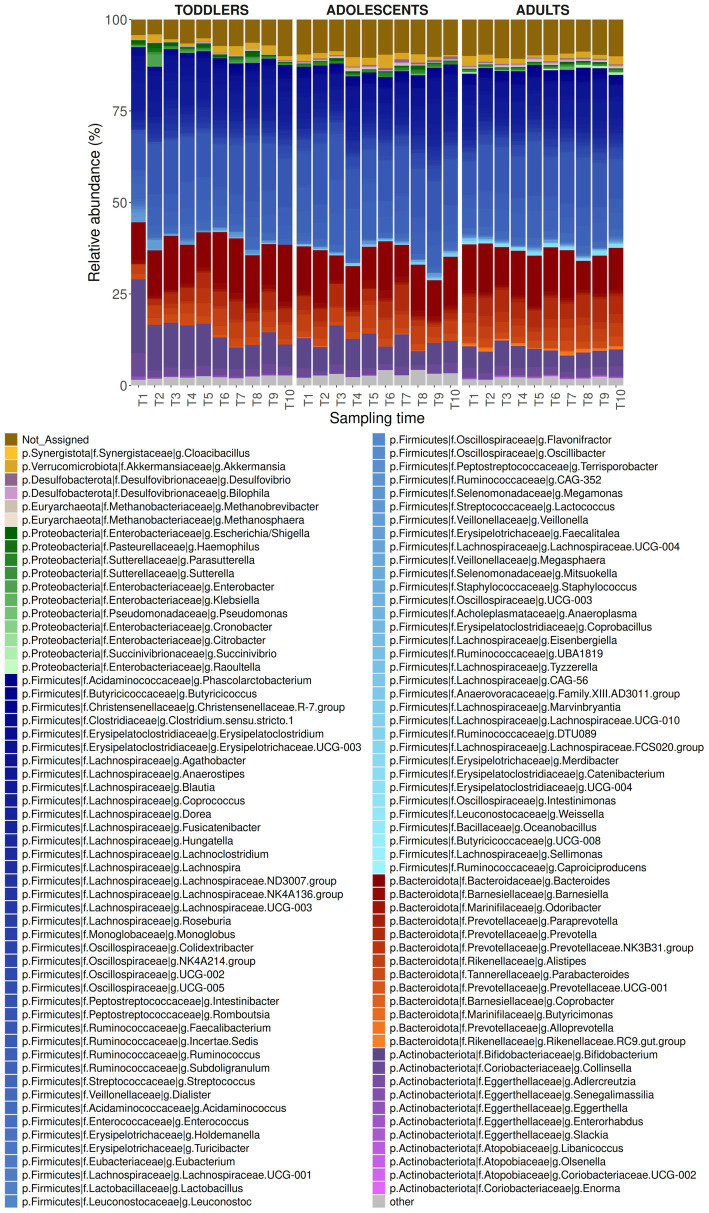
Microbial community composition in fecal samples of toddlers, adolescents, and adults. Relative abundances of bacterial genera (*y*-axis). Each bar represents the mean relative abundance of all individuals from the same age group for each sampling time point (*x*-axis).

Although there is interindividual variation in the relative abundance of different phyla, Firmicutes is the most abundant phylum in all subjects in the three age groups, followed by Bacteroidota and Actinobacteriota and, at lower abundances, Proteobacteria and Verrucomicrobiota (Figure 1) [although current valid names for bacterial phyla are Bacillota (in lieu of Firmicutes), Actinomycetota (in lieu of Actinobacteriota), and Pseudomonadota (in lieu of Proteobacteria), we will use the older names in order to maintain agreement with the database versions used in our analyses].

**Figure 2:**
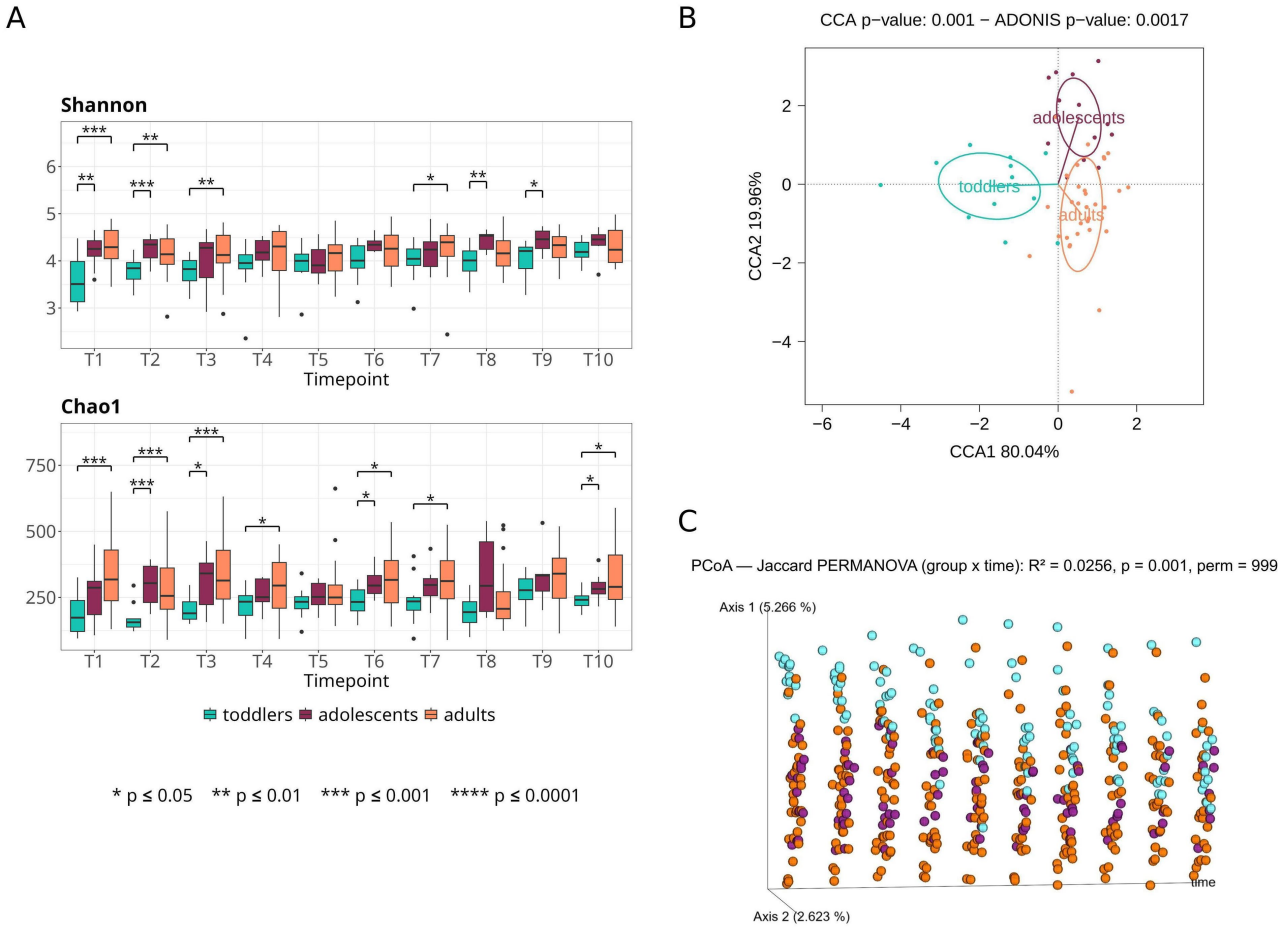
Longitudinal development of gut microbiota diversity and composition at ASV level. **(A)** Shannon diversity and Chao1 richness measurements for each age group during the sampling time, **(B)** CCA and PERMANOVA (Adonis function) based on aggregated data (median relative ASV abundance for all time points of an individual), and **(C)** PCoA based on Jaccard index distances for all samples at each time point.

In terms of alpha diversity computed at the level of ASVs, toddlers have the lowest values for the Shannon index and the Chao1 richness estimator throughout the sampling period. Values for both diversity indexes are mostly significantly lower than those of adolescents and adults during the first three timepoints (Figure 2A; Mann–Whitney *U* test; *p*-value < 0.05. All *p*-values available in Supplementary Table S4), and then increase until becoming similar to those of the other age groups by T4 (32 months of age in average). Adolescents and adults have similar alpha diversity values throughout the sampling period (Mann–Whitney *U* test; *p*-value > 0.05). For the Shannon index, we tested whether diversity values change significantly between the first and the last sampling time points in the different groups (Supplementary Figure S1A). There is a significant change in the microbiota diversity of toddlers between T1 and T10 (Wilcoxon signed-rank test; FDR *p*-value = 0.00293), with an average difference of almost one point in the Shannon index value, whereas the difference in diversity between these points is close to 0 in both adolescents and adults. Accordingly, the T10-T1 difference is significantly higher in toddlers compared to the other two groups (Mann–Whitney *U* test; FDR *p*-value = 0.00121 vs. adolescents and FDR *p*-value = 0.00040 vs. adults). LME models further confirm that Shannon diversity increases with time in toddlers, while in adults and adolescents it remains at very similar values during the sampling period (Supplementary Figure S1B).

In order to visualize whether age drives differences in gut microbiota composition, we performed CCA and PERMANOVA for the three age groups (Figure 2B) and for each pairwise age comparison (Supplementary Table S5) at the levels of ASV, species, genus, and phylum relative abundances. Fecal samples from toddlers cluster away from those of adolescents and adults (PERMANOVA and CCA *p*-value < 0.01) in all pairwise comparisons, except in the “toddlers vs. adolescents” comparison at phylum level (CCA *p*-value = 0.45; PERMANOVA *p*-value = 0.57). When the three groups are considered, the first CCA axis separates toddlers from adolescents and adults, representing 80.04% of the age-associated variability at ASV level, whereas the second axis separates adolescents from adults (19.96% of the variability explained). In this case, CCA and PERMANOVA *p*-values are significant at ASV, species and genus levels, but not at phylum level. Furthermore, to visualize the temporal dynamics within each age group, we performed a PCoA based on Jaccard index distances and plotted the values of the first and second axes against time (Figure 2C). This plot reveals that, while the location of the samples from adolescents and adults is conserved through time, the toddlers’ samples initially occupy higher values on the first axis; however, over time there is a directional change of the toddlers samples towards the adult gut microbiota composition, so that, towards the end of the sampling period, all age groups share similar values on this axis. Analyses based on Bray-Curtis and UniFrac (weighted and unweighted) distances yielded similar results.

**Figure 3:**
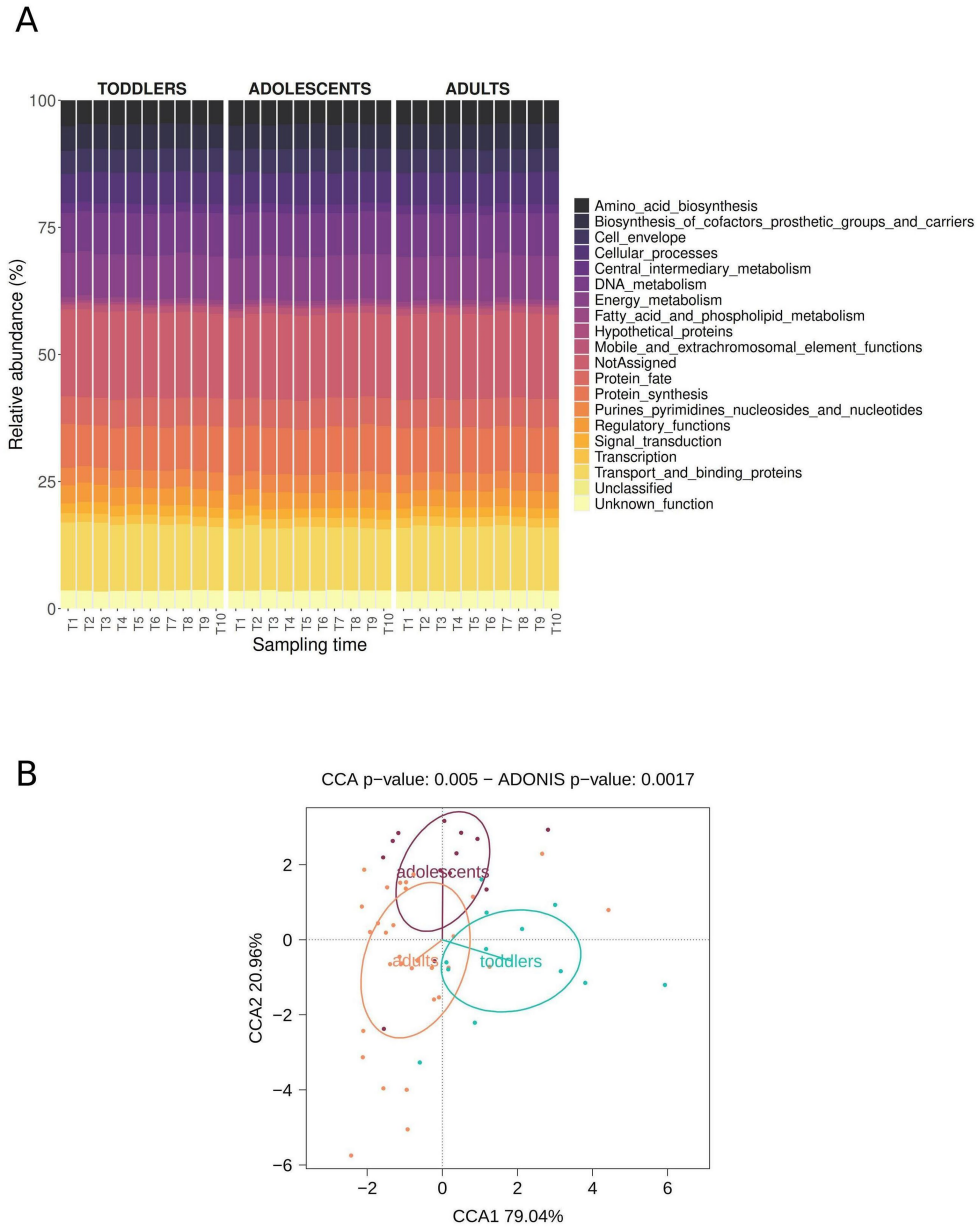
Functional profile in fecal samples of toddlers, adolescents, and adults. **(A)** Relative abundances (*y*-axis) at TIGRFAM main role level for each sampling timepoint (*x*-axis) in each age group. **(B)** CCA and PERMANOVA (Adonis function) based on aggregated data (median relative TIGRFAM subrole abundance for all time points of an individual).

The functional profile of the metagenome, based on the TIGRFAM hierarchical classification, reveals that inter- and intra-individual variation are less pronounced compared to the taxonomic composition (Figure 3A). Functions related to the category (or main role) “transport and binding proteins” are the most abundant across all samples, followed by those related to “protein synthesis,” “protein fate,” “energy metabolism” and “DNA metabolism.” As in the case of the amplicon data, we performed CCA and PERMANOVA for the three age groups and for each pairwise age comparison (Figure 3B; Supplementary Table S5) at the level of TIGRFAM subroles and at the lower level of individual TIGRFAM protein annotations. Samples from the three different age groups cluster away from each other (PERMANOVA and CCA *p*-value < 0.05) in all cases except in the adolescents-adults comparison (PERMANOVA p-value = 0.42 and CCA *p*-value = 0.29 at TIGRFAM protein annotation level; PERMANOVA p-value = 0.94 and CCA *p*-value = 0.64 at TIGRFAM subrole annotation level); therefore, differences between adolescents and adults are less pronounced for function than for taxonomic composition.

### 3.3 Detection of characteristic bacteria for each age group

**Figure 4:**
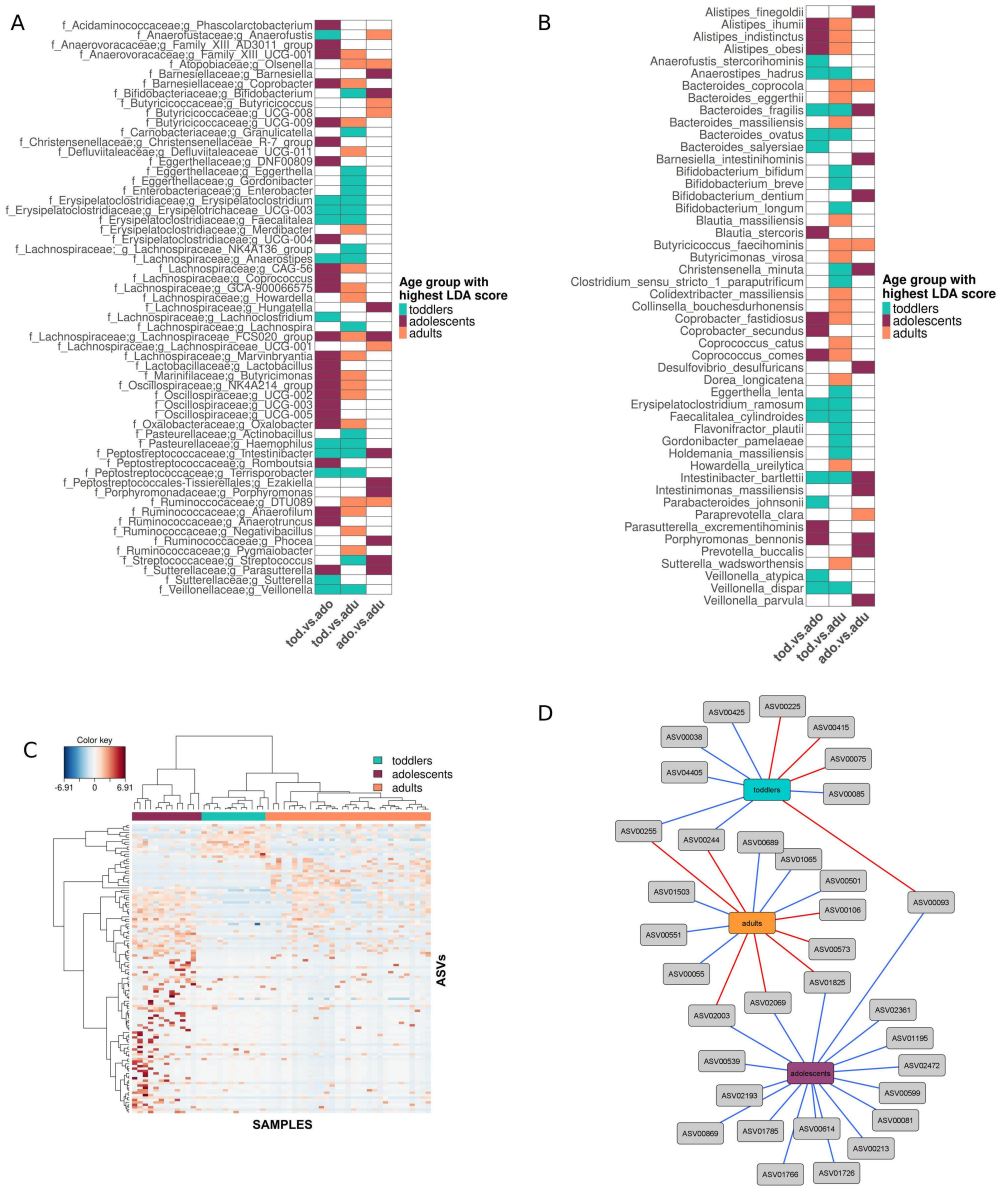
Taxonomic biomarkers of the different age groups. Taxonomic differences between the three age groups showing the age group in which the feature is significantly over-represented according to the LEfSe LDA score (log 10) in each pair-wise comparison (“toddlers *versus* adolescents”, “adolescents *versus* adults” and “toddlers *versus* adults”) at genus **(A)** and species **(B)** levels. **(C)** CIM at ASV level where samples are represented in columns and selected ASVs in rows (10 and 110 ASVs selected on the first and second sPLS-DA components, respectively). The taxonomic identification of the ASVs can be found in Supplementary Table S6A. **(D)** Relevance network of the ASVs selected by sPLS-DA, showing positive (blue) and negative (red) correlations (*r* > 0.4) between ASVs and age groups. The taxonomic identification of the ASVs and their correlation coefficient r with each age group can be found in Supplementary Table S6B.

Pairwise comparisons show that two phyla are significantly over-represented in toddlers compared with adults, Actinobacteriota (LDA score = 4.5; *p*-value = 0.01) and Proteobacteria (LDA score = 3.99; *p*-value = 0.001), while their abundances in adolescents are intermediate. Analyses at the genus and species level (Figures 4A,B, respectively) indicate that these over-representations in toddlers are mainly due to *Eggerthella* (*E. lenta*), *Gordonibacter* (*G. pamelaeae*), and *Bifidobacterium* (*B. longum*, *B. bifidum*, *B. breve*) in the Actinobacteriota, and to *Enterobacter*, *Actinobacillus*, and *Haemophilus* in the Proteobacteria. *Haemophilus* is also over-represented in toddlers when compared with adolescents, indicating that its relative abundance has already decreased by this period. In contrast, *Bifidobacterium* is not over-represented in toddlers compared to adolescents, but rather decreases significantly between adolescents and adults. Within the Actinobacteriota and Proteobacteria there are also some organisms that are under-represented in toddlers, as is the case of *Olsenella,* Eggerthellaceae *DNF00809, Parasutterella* (*P. excrementihominis*) and *Oxalobacter*. *Oxalobacter* is under-represented in toddlers relative to both adolescents and adults, indicating that it increases before adolescence, whereas *Olsenella* is over-represented in adults relative to both toddlers and adolescents, indicating that it increases after this period. On the other hand, the relative abundances of Eggerthellaceae *DNF00809 and Parasutterella* have their highest values in adolescents but are only significantly over-represented in this group relative to toddlers, not to adults.

Within the Firmicutes phylum, there are also several bacterial groups that are over-represented in toddlers with respect to both adolescents and adults, including various members of the Erysipelotrichaceae and Peptostreptococcaceae, as well as *Anaerostipes* (*A. hadrus*) and *Veillonella* (*V. dispar*). Several others still, including *Streptococcus* and two Lachnospiraceae, are over-represented only in relation to adults, indicating that they only decrease significantly after adolescence. On the other hand, many Firmicutes are under-represented in toddlers with respect to adolescents and adults, belonging to the families Anaerovoracaceae, Butyricicoccaceae, Lachnospiraceae, and Oscillospiraceae. There are also numerous taxa that appear to have increased gradually between the toddler years and adulthood, since they occur at low abundances in toddlers with respect to adults, but they do not differ significantly from adolescents in either group. These include members of various families, including the Oscillospiraceae, Defluviitaleaceae, and Erysipelotrichaceae. In contrast, Ruminococcaceae DTU089 and *Butyricicoccus faecihominis* are over-represented in adults with respect to both toddlers and adolescents, but do not differ significantly between adolescents and toddlers, suggesting that they started increasing only after the adolescence period. Finally, there are also some organisms within the Firmicutes having their highest or lowest relative abundances in the adolescents group. These patterns are most pronounced for Lachnospiraceae FCS020 and *Anaerofustis*, which are over- and under-represented, respectively, in relation to both other age groups.

In the Bacteroidota phylum there are no genera over-represented in toddlers, but the species *Bacteroides fragilis* and *B. ovatus* are over-represented relative to both adolescents and adults. Interestingly, *B. fragilis* remains over-represented in adolescents with respect to adults. In contrast, *B. coprocola* is over-represented in adults in relation to toddlers and adolescents, while *B. eggerthii* and *B. massiliensis* are over-represented only in relation to toddlers. In addition, *Coprobacter* (*C. fastidiosus*) and *Butyricimonas*, as well as several species of the genus *Alistipes* (*A. ihumii*, *A. indistinctus*, *A. obesi*) are under-represented in toddlers with respect to both adolescents and adults, while not differing between adults and adolescents, indicating that they have increased to adult-like abundances by the adolescence period. Finally, there are also some Bacteroidota that appear to peak in the adolescents group. This is the case for *Porphyromonas bennonis*, which is significantly over-represented in adolescents relative to both other groups; in addition, *Barnesiella* (*B. intestinihominis*) and the genus *Porphyromonas* also present their higher abundances in adolescents but are only significantly over-represented with respect to adults.

We further analyzed compositional differences among age groups at ASV level. A Clustered Image Map (CIM) based on the ASVs selected on the two sPLS-DA components (10 and 110 ASVs respectively; Supplementary Table S6A) shows that samples from the same age group cluster together (Figure 4C), and similar results were obtained when performing the analysis at genus level. The network in Figure 4D displays relevant correlations (*r* > 0.4; Supplementary Table S6B) between discriminant ASVs and age groups. Toddlers have positive correlations (blue edges) with ASVs annotated as *Veillonella dispar*, *Bifidobacterium bifidum*, *Parabacteroides distasonis*, *Lachnoclostridium*, *Haemophilus*, and Erysipelotrichaceae, with these last two also correlating negatively with adults (red edges). Negative correlations with toddlers involve three ASVs annotated as Oscillospiraceae and one annotated as *Faecalibacterium prausnitzii,* which is positively correlated with adolescents. ASVs positively correlated with adults are annotated in the Ruminococcaceae, Christensenellaceae, and Lachnospiraceae families, in addition to one ASV annotated as *Bacteroides coprocola*. On the other hand, negative correlations with adults include, in addition to the *Haemophilus* and Erysipelotrichaceae ASVs mentioned above, two ASVs in the Ruminococcaceae family and one assigned to *Desulfovibrio desulfuricans* that are positively correlated with adolescents, as well as two ASVs assigned to *Intestinibacter bartlettii* and *Gordonibater pamelaeae*. Finally, adolescents show only positive correlations with various ASVs, mainly annotated within the Firmicutes families Ruminococcaceae and Lachnospiraceae, including two ASVs annotated to *Faecalibacterium prausnitzii* and two to the genus *Lachnoclostridium*. In addition, there are also positive correlations between adolescents and ASVs annotated to the Firmicutes families Lactobacillaceae, Christensenellaceae, Anaerovoracaceae, and Oscillobacteriaceae, and with ASVs annotated to the Bacteroidota *Butyricimonas* and to the above-mentioned Desulfobacterota *D. desulfuricans.*

### 3.4 Detection of characteristic functions for each age group

**Figure 5:**
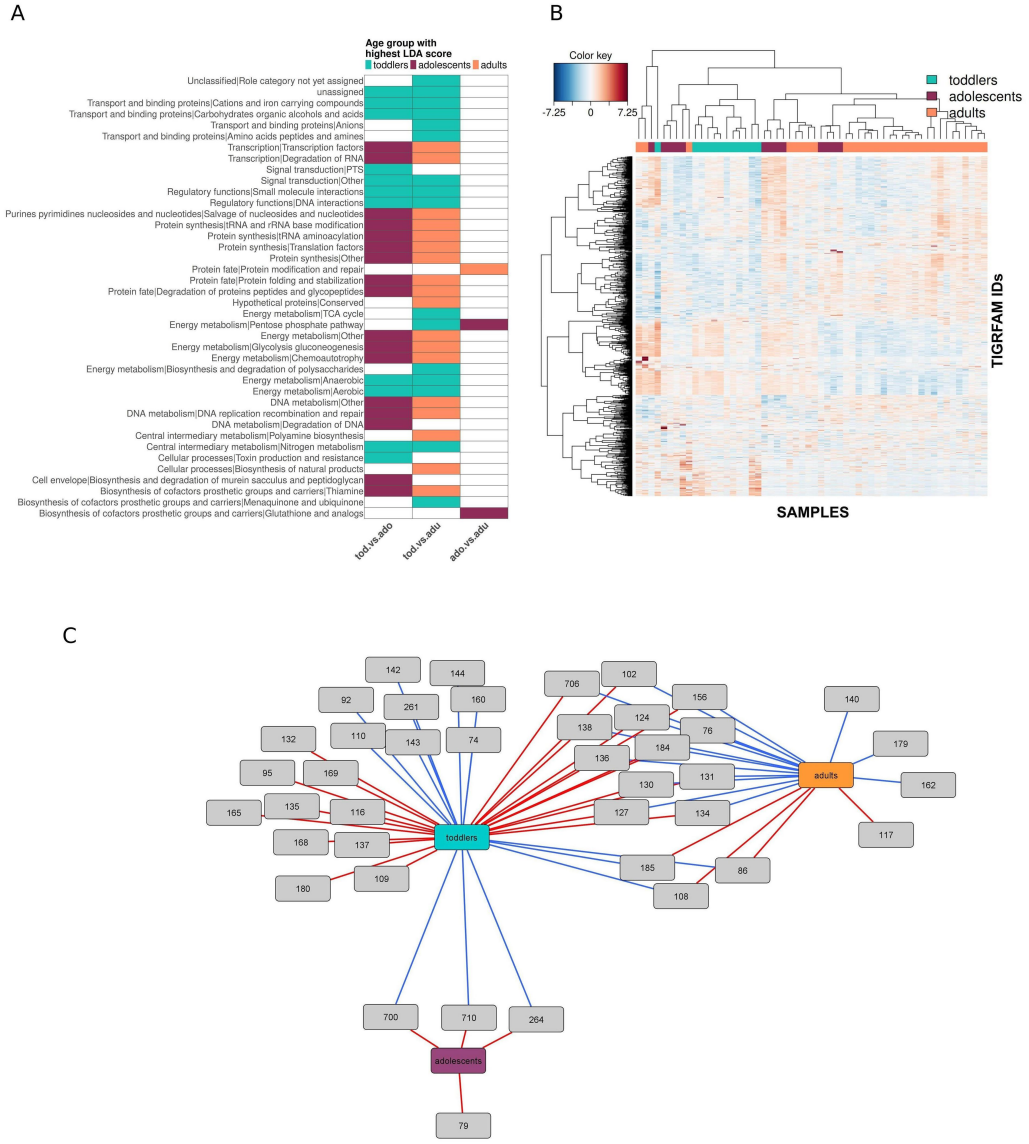
Functional biomarkers of the different age groups. **(A)** TIGRFAM subrole differences between the three age groups showing the age group in which the subrole is significantly over-represented according to the LEfSe LDA score (log 10) in each pair-wise comparison (“toddlers *versus* adolescents”, “adolescents *versus* adults”, and “toddlers *versus* adults”). **(B)** CIM at TIGRFAM level where samples are represented in columns and selected TIGRFAMs in rows (300, 210, 240, and 280 features selected on each sPLS-DA component). The TIGRFAM description, functional subrole and category can be found in Supplementary Table S7A. **(C)** Relevance network of TIGRFAM subroles, showing positive (blue) and negative (red) correlations (*r* > 0.3) between subroles and age groups. The name and functional category of the subroles and their correlation coefficient r with each age group can be found in Supplementary Table S7B.

At functional level, Figure 5A shows an enrichment in toddlers of various subroles belonging to diverse functional categories with respect to both adolescents and adults. These include subroles within the “energy metabolism,” “regulatory functions,” “transport and binding proteins”, “central intermediary metabolism”, and “signal transduction” categories. In addition, the “pentose phosphate pathway” within “energy metabolism” is enriched in toddlers only in relation to adults, suggesting that it decreased after adolescence. There are also several subroles that appear to have decreased gradually between the toddler years and adulthood, since they occur at high abundances in toddlers with respect to adults, but they do not differ significantly from adolescents in either group. These include subroles within the “biosynthesis of cofactors, prosthetic groups and carriers”, “energy metabolism”, and “transport and binding proteins” categories. In contrast, there are numerous subroles that rather increase in adolescence and endure in adulthood. These are distributed across a wide variety of functional categories, including “biosynthesis of cofactors, prosthetic groups and carriers”, “energy metabolism,” “DNA metabolism,” “purines, pyrimidines, nucleosides and nucleotides”, “transcription”, “protein synthesis”, and “protein fate.”

A CIM based on the TIGRFAM annotations selected on the first four components obtained through sPLS-DA (300, 210, 240, and 280 features selected respectively; Supplementary Table S7A) shows that samples from different age groups are less clearly separated at functional than at taxonomical level. Samples from toddlers and adults are mainly represented in single clusters, but samples from adolescents can be found in various clusters also containing samples from the other age groups (Figure 5B). The network in Figure 5C displays relevant correlations (*r* > 0.4; Supplementary Table S7B) between discriminant TIGRFAM subroles and age groups. Toddlers have positive correlations with subroles within a variety of TIGRFAM categories, including “amino acid biosynthesis”, “biosynthesis of cofactors, prosthetic groups and carriers”, “cellular processes”, “protein synthesis”, “energy metabolism”, “transport and binding proteins”, “regulatory functions”, and “signal transduction.” Some of these subroles are negatively correlated with adolescents or adults, including those related to signal transduction and regulatory functions in the first case, and to aerobic energy metabolism and glutathione biosynthesis in the second. Subroles that are negatively correlated with toddlers mainly belong to “transcription”, “protein synthesis”, “protein fate”, “energy metabolism”, “DNA metabolism”, and “purines, pyrimidines, nucleosides and nucleotides”. Many of these subroles, in particular those related to DNA, RNA, and protein degradation, are positively correlated with adults. In addition, adults also have positive correlations with functions in “protein fate”, “central intermediary metabolism”, and “biosynthesis of cofactors, prosthetic groups and carriers”, as well as a negative correlation with the pentose phosphate pathway within “energy metabolism”. Finally, there are no functional subroles positively correlated with adolescents, and only four are negatively correlated with this age group, *i. e.* the above-mentioned subroles that are positively correlated with toddlers and a fourth one belonging to “biosynthesis of cofactors, prosthetic groups and carriers”.

### 3.5 Relating taxonomic and functional signatures of the microbiome

**Figure 6:**
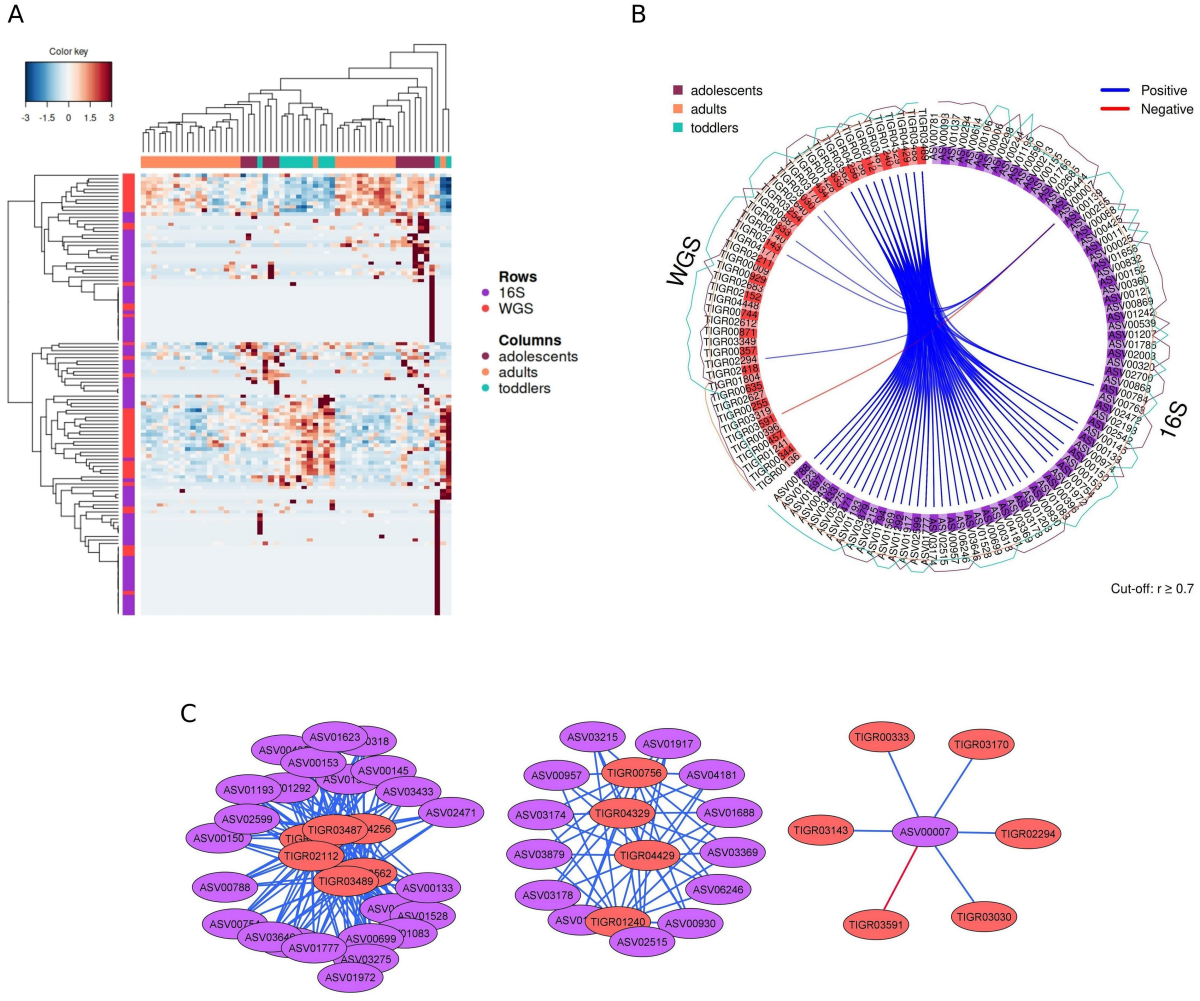
Multiomics integrative analysis. **(A)** CIM of the multiomics molecular signature, where samples are represented in rows and ASVs and TIGRFAMs selected on the first four sPLS-DA components are represented in columns. The identification of the ASV and TIGRFAM features can be found in Supplementary Table S8A. **(B)** Circos plot displaying the positive (blue) and negative (red) correlations (*r* > 0.7) between selected features, showing the age group in which each of the features has a greater presence. **(C)** Relevance network visualization showing positive (blue) and negative (red) correlations between selected ASVs and TIGRFAMs. The annotation of the features and their correlation coefficient *r* can be found in Supplementary Table S8B.

In addition to looking for taxonomic and functional biomarkers in the 16S rRNA gene amplicon and metagenome data sets separately, we tried to identify the relationships between these biomarkers using DIABLO, a multivariate integrative classification method that seeks for molecular signatures across different data types. The CIM based on the molecular signatures identified in the first four components (7, 30, 30, and 12 from the 16S rRNA gene amplicon data, and 30, 5, 5, and 5 from the metagenome data; Supplementary Table S8A) shows that most samples from each age group are contained in two or three distinct clusters, each containing mostly samples from the same group (Figure 6A). The *Circos* plot in Figure 6B highlights the mainly positive correlations between ‘omics features (Pearson’s correlation coefficient >0.7; Supplementary Table S8B), showing the relative abundance of each feature in the three age groups (ASV and TIGRFAM IDs and their associated taxa and subroles, as well as the corresponding age group in which the relative abundance of each feature is highest, are shown in Supplementary Table S8A); most of the features are enriched in toddlers and/or adolescents. The network displaying the connections between the most relevant selected ASVs and TIGRFAMs separates into three subnetworks (Figure 6C and Supplementary Table S8B, Pearson’s correlation coefficient >0.7). The first subnetwork (left) contains 6 TIGRFAMs associated with toddlers encoding three different CRISPR-associated proteins as well as functions related to electron transport and detoxification. These functions positively correlate with 24 ASVs belonging mostly to the families Prevotellaceae, Bacteroidaceae, Lachnospiraceae, Oscillospiraceae and Erysipelotrichaceae, as well as to the species *Phascolarctobacterium succinatutens*, *Veillonella atypica*, and *Butyricicoccus faecihominis*. The second subnetwork (center) contains 4 TIGRFAMs associated with adolescents encoding functions related to CRISPR systems, protein interactions, and the central intermediary metabolism. These functions positively correlate with 13 ASVs, some of which also belong to the Prevotellaceae, Bacteroidaceae, Oscillospiraceae and Erysipelotrichaceae, while others belong to the families Anaerovoracaceae, Peptococcaceae and Ruminococcaceae, and the genera *Alistipes* and *Veillonella*. The third subnetwork (right) includes a single ASV belonging to an unassigned *Bifidobacterium* (ASV00007) that is associated with toddlers. This *Bifidobacterium* ASV correlates positively with functions related to deoxyribonucleotide metabolism, transport and binding proteins, and chemotaxis and motility, and negatively with a TIGRFAM involved in degradation of RNA.

Furthermore, we checked whether the taxonomical annotations of metagenomic reads supported the associations between taxa and functions indicated by DIABLO. Although most of the ORFs corresponding to the TIGRFAMs present in the DIABLO networks were not taxonomically annotated, we could confirm some of the associations. In particular, regarding the TIGRFAMs positively associated with *Bifidobacterium* ASV00007 in the third subnetwork, reads mapping to ORFs annotated as TIGR00333 (nrdl protein, subrole “2-deoxyribonucleotide metabolism”) were present only in toddlers and adolescents and were mostly assigned to *Bifidobacterium*. In contrast, reads mapping to ORFs annotated as TIGR03591 (polyribonucleotide nucleotidyltransferase, subrole “degradation of RNA”), negatively associated with *Bifidobacterium* ASV00007 and over-represented in adults (Supplementary Table S8), were mainly assigned to *Faecalibacterium* (data not shown).

### 3.6 Temporal stability of the microbiota at different ages

**Figure 7:**
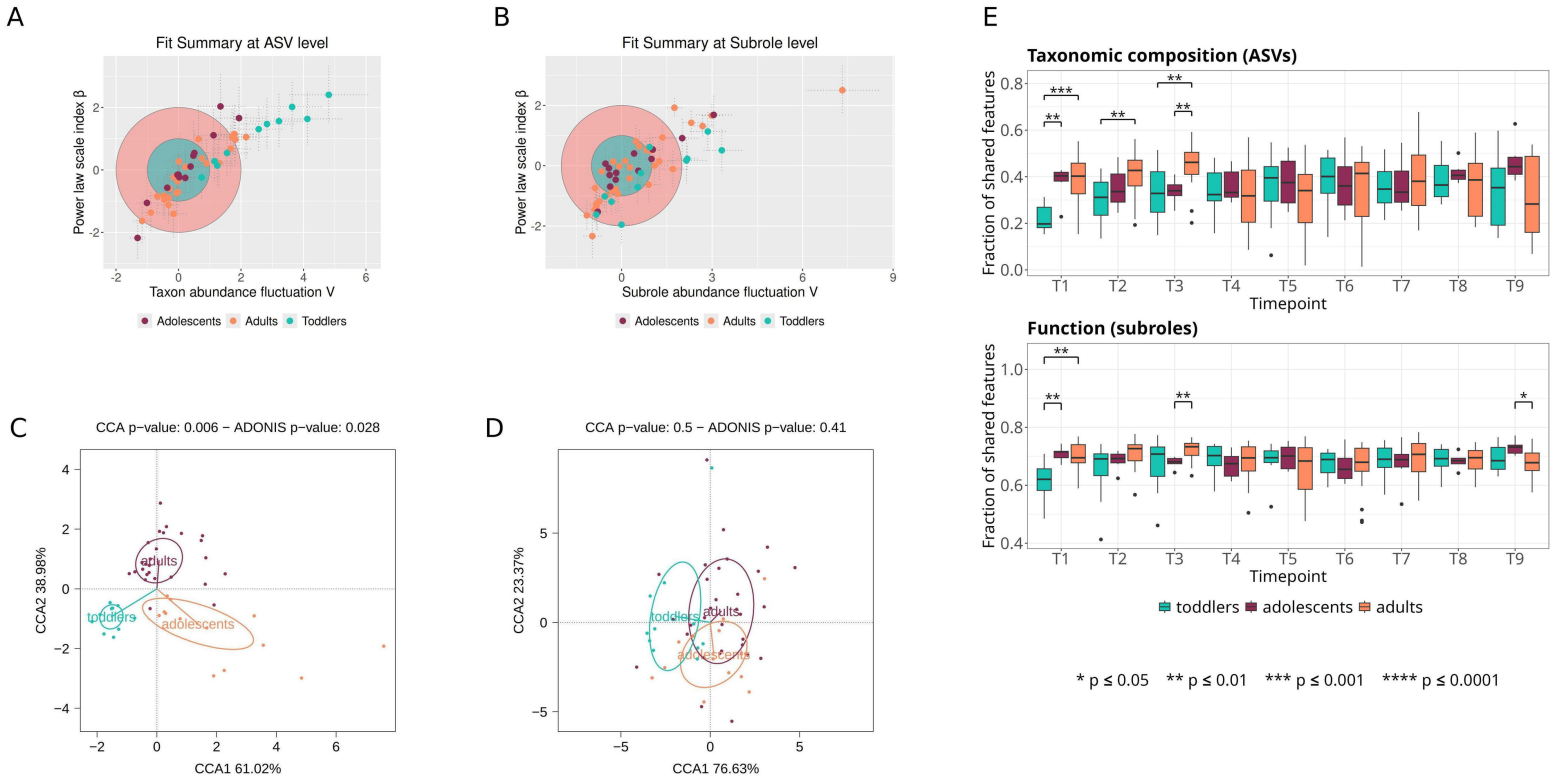
Temporal stability of the microbiota. **(A–D)** Time series analysis with *complexCruncher.* **(A, B)** Plots of feature variability (*V*) in the *x*-axis and Taylor’s law scale index (*β*) in the *y*-axis, both in standard deviation units, for ASVs **(A)** and functional subroles **(B)**; the pink circle corresponds to the region containing 68% of the adult subjects (taken as reference group) and the blue circle corresponds to the 98% region. Dashed lines represent the error of the calculated parameters for each subject. **(C, D)** CCA and PERMANOVA (Adonis function) based on rank stability index (RSI) values, quantifying feature stability over time in each age group for ASVs **(C)** and subroles **(D)**. **(E)** Jaccard index for each consecutive sample pair from the same subject. Each box represents the mean Jaccard index for each set of consecutive sample pairs in each age group at ASV level (top) and TIGRFAM level (bottom); lines connect the means of each box.

Based on *complexCruncher* (Martí et al., 2017) analyses of 16S rRNA amplicon data we can see that toddlers are the most unstable group (Figure 7A), while adolescents and adults have similar distributions of *V* and *β*, which, respectively, represent taxon abundance fluctuations over time and the scale index of the power law relating taxon abundance mean and dispersion. The CCA and PERMANOVA based on RSI values show that the three age groups cluster away from each other (Figure 7C), with adolescents showing the most dispersion among individuals. However, the same analysis with metagenome data (at subrole level) does not show a significant overall difference between the different age groups in terms of stability (Figures 7B,D).

In addition, to further examine the stability of the microbiota over time within the different age groups, we analyzed the amount of change per unit of time throughout the sampling period. For this purpose, we calculated for all pairs of consecutive samples from the same subject the Jaccard index, a beta-diversity metric that indicates the fraction of shared features between samples, both at compositional and functional levels, considering ASV and TIGRFAM annotations, respectively. Stability in all three age groups was lower at the compositional level than at the functional level (Figure 7E). At both the compositional and functional levels, adolescents and adults had similar values of the Jaccard index throughout the sampling period. The only exception is at interval T3-T4, for which adults have a high Jaccard index value compared to all other intervals. In contrast, toddlers had a significantly lower stability than adolescents and adults during the first-time interval (Mann–Whitney *U* test, *p*-value < 0.01 for the T1-T2 comparison at both levels. All *p*-values available in Supplementary Table S9), with the fraction of shared features between consecutive samples increasing after that. This indicates that both the taxonomic and the functional composition of the microbiota stabilize during the toddler period.

**Figure 8:**
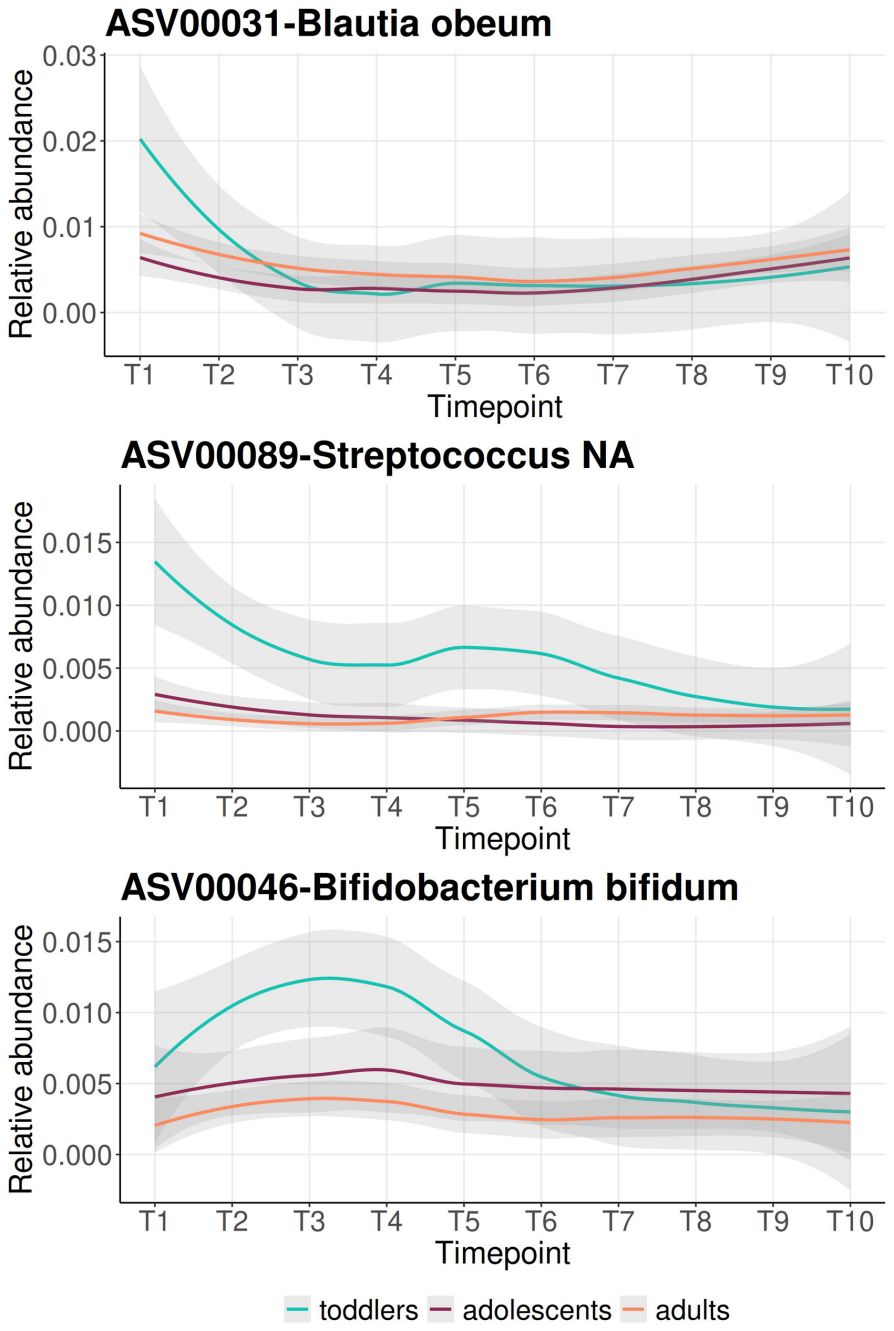
Feature volatility plots for ASVs that are predictive of the time point. Longitudinal relative abundances (*y*-axis) at each time point (*x*-axis) are represented for ASV00046 (*Bifidobacterium bifidum*).

Furthermore, in order to detect temporal trends of taxon variability within age groups we used a supervised learning regressor within the q2-longitudinal pipeline included in QIIME 2 (Bokulich et al., 2018). This allowed us to identify several cases in which an initial over-representation of an ASV in toddlers disappeared during the sampling period (Figure 8). This was the case of ASV00031 (*Blautia obeum*), ASV00089 (*Streptococcus* NA), and ASV00046 (*Bifidobacterium bifidum*). ASV00031 was found at very low abundance in adolescents and adults throughout the sampling period, while in toddlers it had the highest abundance at T1 but dropped close to 0% at T2 (24 months of age in average; Figure 8A). ASV00089 was also nearly absent in adolescents and adults, while in toddlers it was highest at T1, decreased from T1 to T2, and then remained at low levels until T9 (4.17 years of age in average), at which point it practically disappeared (Figure 8B). Also noteworthy is the case of ASV00046, which appeared in similar relative abundances in the three groups at T1 (although highest in toddlers and lowest in adults) and then rose in toddlers until T4 (32 months of age in average), to decrease to values similar to those of adults and adolescents by T5 (36 months of age in average; Figure 8C).

### 3.7 Changes in the gut microbiota after weaning in toddlers

**Figure 9:**
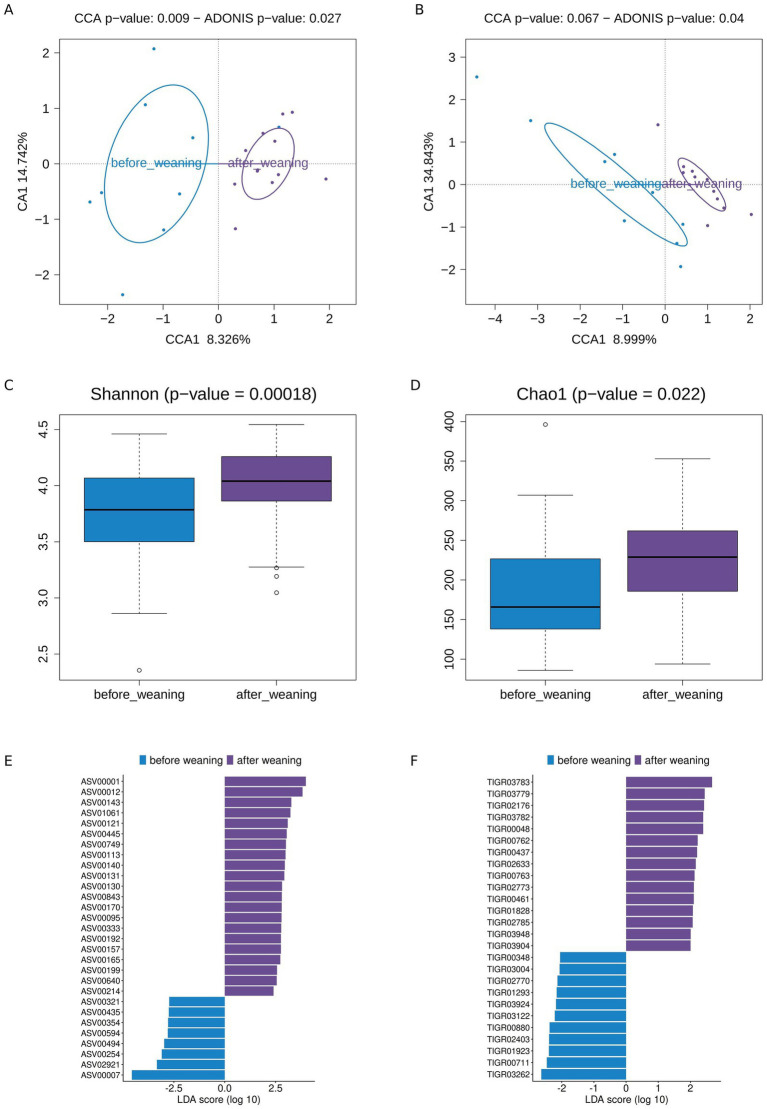
Weaning analysis. CCA at the level of **(A)** ASV and **(B)** TIGRFAM relative abundances, based on aggregated data (median relative feature abundance for all time points of an individual before or after weaning). Alpha diversity of samples from toddlers before and after weaning based on **(C)** Shannon index and **(D)** Chao1. LEfSe analysis of differences between samples from toddlers before and after weaning at **(E)** ASV and **(F)** TIGRFAM levels.

Among toddlers, most participants (9/12) were still consuming breast milk when they entered the study and were weaned during its course. To investigate how the cessation of breast milk feeding impacted the composition and function of the toddlers’ microbiome, we compared aggregated samples before and after weaning (*N* = 27 and *N* = 72 respectively; Supplementary Table S1). CCA and PERMANOVA showed that samples before and after weaning differed significantly at ASV level (Figure 9A; *p*-value < 0.05) and almost significantly at TIGRFAM level (Figure 9B; PERMANOVA and CCA *p*-values = 0.053 and 0.056 respectively). In addition, diversity increases after weaning (Mann–Whitney *U* test: Shannon index *p*-value = 0.00039; Chao1 *p*-value = 0.00025; Figures 9C,D respectively).

At taxonomic level, Figure 9E shows that before weaning there is an over-representation of three ASVs of *Veillonella*, two of *Bifidobacterium* (including ASV00007), and single ASVs of *Gemella sanguinis*, Clostridia UCG-014 and *Erysipelatoclostridium ramosum*. After weaning, ASVs annotated as *Ralstonia*, *Bacteroides*, *Odoribacter splanchnicus*, *Blautia*, *Lachnospira pectinoschiza*, and the Clostridia vadinBB60 group are over-represented, as well as 13 ASVs in the Oscillospiraceae family.

At functional level, TIGRFAMs over-represented before weaning (Figure 9F) belong to subroles in the “cellular processes,” “protein fate,” “transport and binding proteins”, “biosynthesis of cofactors, prosthetic groups, and carriers”, and “DNA metabolism” categories. Most of these subroles were also over-represented in toddlers in comparison to adolescents and adults. After weaning, over-represented TIGRFAMs mostly belong to different subroles within the same functional categories; in addition, three TIGRFAMs belong to energy metabolism subroles. Some of these subroles, related to DNA replication, protein degradation, and energy metabolism, were under-represented in toddlers compared to adolescents and adults.

### 3.8 Changes in the gut microbiota after menarche in female adolescents

**Figure 10:**
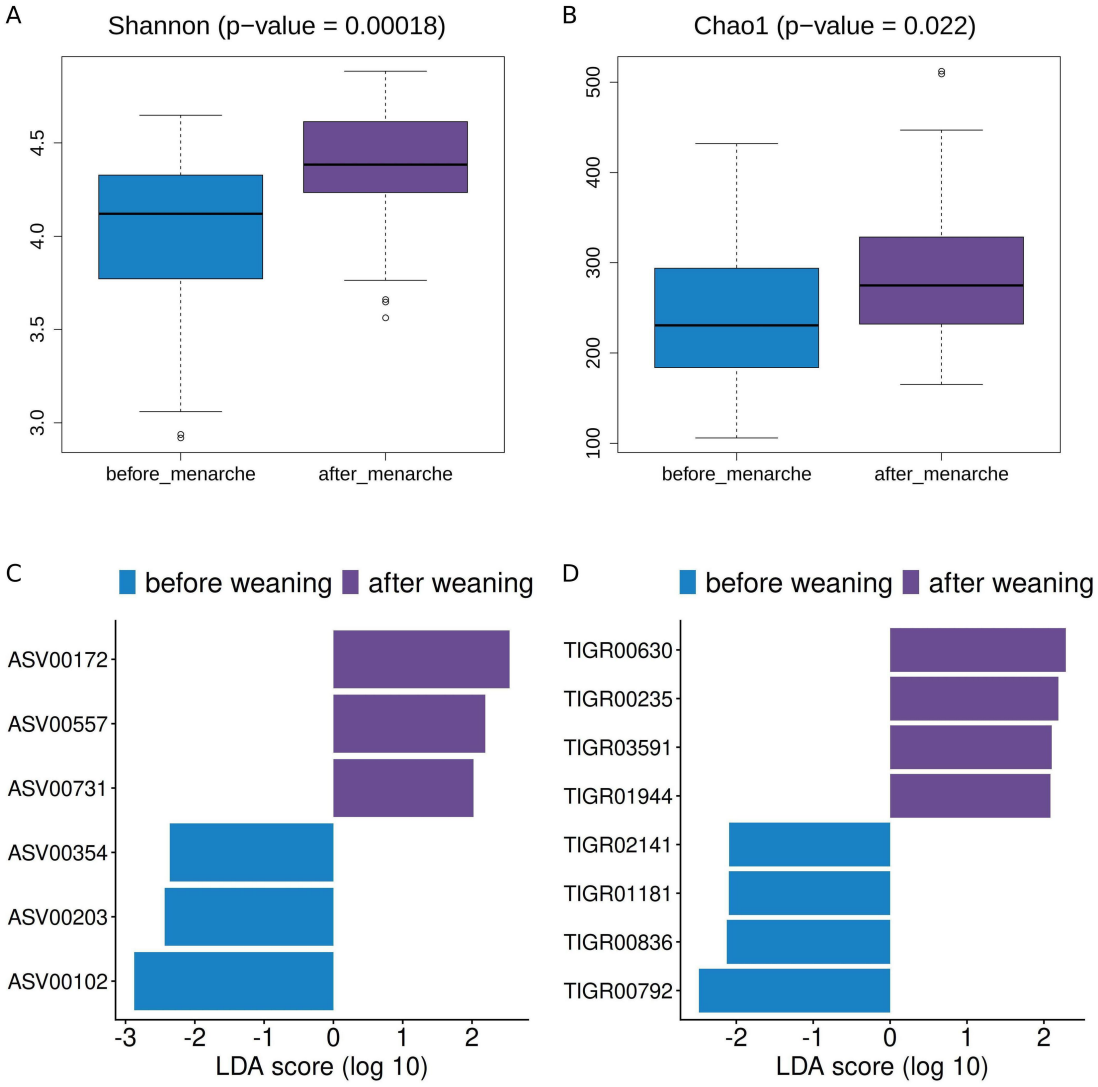
Menarche analysis. Alpha diversity of samples from female adolescents before and after menarche based on **(A)** Shannon index and **(B)** Chao1. LEfSe analysis of differences between samples of adolescents before and after menarche at **(C)** ASV and **(D)** TIGRFAM levels.

In the adolescents’ group, most female participants (7/9) had not undergone menarche when they entered the study and most of them (6/7) underwent this event during its course. Since changes in hormone levels associated to menarche could affect the composition and function of the gut microbiota, we analyzed separately the aggregated samples of female adolescents before (*N* = 18) and after (*N* = 18) the menarche event (Supplementary Table S1). CCA and PERMANOVA showed that samples before and after menarche did not differ significantly from each other neither at the taxonomic nor at the functional level (*p*-values > 0.05 for ASV, species, genus, TIGRFAM and subrole). However, in terms of diversity, both the Shannon index and the Chao1 estimators increased significantly after menarche (Figures 10A,B; *p*-values = 0.00018 and 0.022, respectively).

At taxonomic level, Figure 10C shows that ASVs of *Coprococcus catus, Clostridium* sensu stricto and *Veillonella*, all within the phylum Firmicutes, are over-represented before menarche, while ASVs annotated as *Butyricicoccus faecihominis*, *Adlercreutzia equolifaciens* and an unassigned Desulfovibrionaceae are over-represented after menarche. At functional level (Figure 10D), TIGRFAMs that are over-represented before menarche belong mainly in the “transport and binding proteins” category, with a single TIGRFAM belonging to the “cell envelope” category. A more heterogeneous group of TIGRFAM annotations are over-represented after menarche, belonging to a variety of functional categories and including functions related to DNA replication, salvage of nucleosides and nucleotides, degradation of RNA, and electron transport.

## 4 Discussion

In the present study we have analyzed the changes that occur in the intestinal microbiota with development to better characterize and distinguish the healthy microbiota of young children, adolescents, and adults. To this end, we have compared diversity, composition, functional profile, stability, and dynamics of the microbiota in these three age groups, and we have identified biomarkers from each. Moreover, although our study was not specifically designed to assess their effect, we have also evaluated the potential impact on the microbiome of two main developmental events encompassed by the age ranges sampled in our study, namely weaning and menarche; however, the analyses of the effects of these two events are exploratory due to the small sample sizes and consequent limitation in power.

The microbiota of toddlers at the beginning of the study is significantly less diverse than those of adolescents and adults, but diversity increases to levels similar to those of these age groups by T4, when toddlers are 32 months of age in average. Diversity is higher after weaning, but some toddlers had not yet been weaned by T4, suggesting that other factors may also contribute to this increase.

In terms of taxonomic composition, differences among toddlers and adults are still substantial enough to be detected even at the level of phyla, with an over-representation of Actinobacteriota and Proteobacteria. Within the latter, the high abundances of enteric bacteria characteristic of infants are no longer present, and two of the three over-represented genera are potential pathogens belonging to the Pasteurellaceae (*Haemophilus* and *Actinobacillus*). The fact that microbiota composition in a subgroup of samples from toddlers was influenced by breast milk consumption clearly contributed significantly to many of the differences observed when the entire group of toddlers was compared to adolescents and adults. Indeed, it is mostly the taxa that are under- or over-represented before weaning that also appear as under- or over-represented in toddlers as a whole when these are compared to the other age groups. This is the case in particular for under-represented members of the family Oscillospiraceae and for several important over-represented taxa such as *Bifidobacterium*, *Veillonella*, and *Erysipelatoclostridium*.

*Bifidobacterium* is one of the main taxa that distinguish toddlers from adults, but, interestingly, we cannot consider it uniquely characteristic of this age group since it is still abundant in adolescents. Nevertheless, as expected, *Bifidobacterium* is favored by breast milk intake in toddlers, since it is more abundant before weaning, in accordance with the fact that many species of this genus are adapted to the utilization of breast milk oligosaccharides. The high abundance in toddlers of lactate producers such as *Bifidobacterium* may contribute to the over-representation at this stage of lactate-utilizing taxa such as *Anaerostipes* and *Veillonella* (Duncan et al., 2004; Sato et al., 2008; Reichardt et al., 2014). Experiments in anaerobic fermentors growing human colonic bacteria have shown that lactate accumulation results in major shifts in microbiota composition; while the Bacteroidota and most Firmicutes, including the main butyrate producers of the adult gut, decrease, *Anaerostipes* and *Veillonella* as well as the Actinobacteria and Proteobacteria increase (Wang et al., 2020). Lactate may therefore be an important factor favoring the growth of these over-represented taxa in toddlers.

The fact that many of the toddlers still complemented their diet with breast milk likely also contributed to the over-representation of other taxa. For instance, *Erysipelatoclostridium* has been positively associated with dairy intake in both adults and toddlers (Smith-Brown et al., 2016; Swarte et al., 2020), and we find it over-represented in toddlers in relation to the other age groups, and, within these, in those that have not been weaned. Other dietary factors may further select for this taxon. Due to their growth-related needs, and to the fact that they oxidize fats more readily (Kostyak et al., 2007), young children require higher energy and lipid intakes per kilogram of weight than adults, as well as a higher percentage of fat relative to total energy intake. The class Erysipelotrichia is enriched in the intestines of obese humans and mice and *E. ramosum* has been shown to increase body weight, body fat and food efficiency in mice fed a high fat diet (Woting et al., 2014). Therefore, *Erysipelatoclostridium* and other Erysipelotrichaceae may be favored in young children, potentially contributing to increased lipid absorption and food efficiency.

In addition, it is also noteworthy that for some genera, although overall relative abundance does not differ in toddlers, some particular species are over- or under-represented in this group, suggesting age specialization at the species level. This is the case for *Bacteroides*, in which *B. fragilis* and *B. ovatus* are over-represented while *B. coprocola*, *B. eggerthii*, and *B. massiliensis* are under-represented in relation to adults. Previous studies have reported that *B. fragilis* is the most abundant species in infants until 10 months of age (Stewart et al., 2018). Here, we further detect that *B. fragilis* is still significantly over-represented in toddlers in comparison to the other age groups, and remains so in adolescents in comparison to adults, where it is found at very low levels. This suggests that *B. fragilis* is an early colonizer of the human gut that fares better in younger individuals and decreases in relative abundance during a protracted extension of time, continuing after the adolescence period.

The intestinal microbiota of adolescents appears to be in an intermediate stage of development in terms of taxonomic composition, as shown by the high relative abundance of bacteria such as *Bifidobacterium* and *Bacteroides fragilis*, which are highly abundant in infants and toddlers (Hopkins et al., 2001; Tannock et al., 2013; Stewart et al., 2018). Moreover, the fact that we are able to analyze children, adolescents and adults within the same study allows us to define when some compositional changes occur relative to adolescence. Some bacteria have increased or decreased in abundance before this stage, while others have increased or decreased between adolescence and adulthood. For instance, our results indicate that some microaerophilic or facultatively anaerobic bacteria, such as *Veillonella* and the potentially pathogenic *Haemophilus*, which causes infections mainly in childhood, have already decreased in abundance by adolescence. On the other hand, some bacteria of the intestinal microbiota that are considered indicators of health in adulthood have increased to adult-like levels by adolescence, as is the case of *Faecalibacterium prausnitzii*, one of the most important butyrate-producing bacteria, the decrease of which in the intestinal tract has been related to the triggering of inflammatory processes (Ferreira-Halder et al., 2017). In contrast, bacteria that change in abundance after adolescence include the lactic acid bacteria *Bifidobacterium* and *Streptococcus* as well as *B. fragilis* and various Lachnospiraceae, which decrease after this period, as well as Ruminococcaceae DTU089 and *Butyricicoccus faecihominis*, which rather increase between adolescence and adulthood. Interestingly, different studies have shown that the relative abundances of various members of the family Ruminococcaceae increase with age, being significantly higher in the elderly population and especially in high longevity people (>90 years old) (Biagi et al., 2016; Kong et al., 2016; Yang et al., 2020), suggesting that members of this family of butyrate-producers may continue to increase throughout life. Finally, in some cases it is during adolescence that some groups of bacteria reach their peak of abundance, suggesting that these organisms may be particularly adapted to this period. Such bacteria include Lachnospiraceae FCS020 and *Porphyromonas bennonis*. In adults, the abundance of Lachnospiraceae FCS020 has been correlated with exercise training (Verheggen et al., 2021) and with a specific distribution of lipoprotein particle types (Vojinovic et al., 2019), suggesting that the high abundance of these bacteria in adolescents may relate to their activity patterns or to specificities of their lipid metabolism.

Since we know that major hormonal changes can affect the gut microbiota (Koren et al., 2012), we have also attempted an exploratory analysis of the differences in the gut microbiota before and after menarche in adolescent girls. Despite the small number of samples, we have seen an increase in diversity after menarche, as well as moderate changes in composition. Several of the observed differences suggest that the microbiota before menarche may still harbor some features detected in young children, such as an over-representation of *Veillonella*, whereas the over-representation of some taxa characteristic of adults, such as *Butyricicoccus faecihominis,* appears after menarche. However, more studies are needed to understand the changes that occur in the intestinal microbiota around this period.

In terms of functional composition, the differences between the age groups are less pronounced, with toddlers again presenting the greatest difference with respect to the other two. In a similar manner to what is seen in terms of taxonomic composition, many functions that are under or over-represented in toddlers as a whole in relation to adolescents and adults are also under or over-represented in toddlers before weaning. This includes, for instance, functions related to DNA replication and protein degradation, which have higher relative abundances in adolescents and adults than in toddlers and also seem to increase among toddlers after weaning. Our exploratory analyses therefore suggest that breast milk contributes significantly to shaping not only the taxonomic composition but also the functionality of the microbiota of toddlers, and that weaning contributes to generate a functional profile more similar to that in the older age groups. This is in agreement with recent metabolomic analyses showing that breastfeeding is the most dominant factor associated with gut metabolite levels, impacting metabolite concentrations up to 30 months of age (Aatsinki et al., 2025). However, adolescents and adults also have higher relative abundances of other functions that we do not detect to increase after weaning, particularly in functional categories related to genetic information processing such as “DNA metabolism,” “transcription,” “protein synthesis” and “protein fate,” which likely increase later in childhood.

In contrast, toddlers are enriched in several energy metabolism functions, including, but not limited to, functions related to aerobic respiration. Some of these functions are significantly reduced only after adolescence, such as “biosynthesis and degradation of polysaccharides”, “pentose phosphate pathway” and “TCA cycle.” Therefore, also at a functional level, adolescence seems to represent a stage of transition towards an adult microbiome, where some functions have attained adult-like relative abundances whereas others are still present at the levels seen in young children. In particular, our exploratory analyses suggest that adolescent girls before menarche are enriched in some transport and binding functions associated with toddlers, whereas after menarche we see the increase of functions under-represented in toddlers related to “DNA metabolism” and “transcription.”

Network analyses allowed us to identify bacteria whose abundance correlates with some of the functions enriched in the different age groups. In particular, an abundant *Bifidobacterium* ASV (ASV00007) was linked with several toddler-associated TIGRFAMs. Taxonomic annotation of metagenomic reads also indicated that *Bifidobacterium* contributed to the TIGRFAMs over-represented in this group. Interestingly, *Bifidobacterium* has been shown to be among the taxa whose presence in infant samples correlates with age-associated metabolites (Wu et al., 2024). In addition, *Veillonella*, *Alistipes*, as well as members of the Lachnospiraceae and Prevotellaceae families, are also prominent both in our networks of interaction with TIGRFAMs and in the microbe-metabolite correlations detected by Wu et al. (2024). This might suggest that these bacteria are especially relevant in contributing specific functions to the gut ecosystem.

Studies of the long-term stability of the microbiota are rare due to the difficulty in following the same individual through long periods of time. Here, we have been able to analyze stability throughout a 3-year period in toddlers, adolescents and adults of the same population, at the level of taxonomic composition and functional profile. Our analyses show that the stability of the microbiota is lower at taxonomic than at functional level in all three age groups, indicating the existence of functional redundancy through time. Overall, when the three age groups were considered over the entire period of sampling, *complexCruncher* detected that toddlers were significantly more unstable than the other two groups only at the level of taxonomic composition (Figure 7). Further, local stability analyses revealed that, for both taxonomic composition and functional profile, it was only the first time interval in toddlers (T1-T2, between 21 and 24 months in average) that showed a drastically lower stability, while after this point stability levels were similar to those of adolescents and adults. A supervised learning regressor approach allowed us to identify those ASVs that changed drastically in abundance in toddlers between T1 and T2 (Figure 8), belonging to *Blautia obeum* and *Streptococcus*. These results indicate that the microbiota stabilizes at around 2 years of age, after which the overall amount of change per unit of time in toddlers is not significantly higher than that in the other age groups, neither in taxonomic composition, nor in functional profile.

Taken together, our results suggest that the intestinal microbiota stabilizes early on during childhood in terms of large-scale, rapid changes in diversity, composition, and functional profile. Our results also indicate a very substantial impact of weaning on the diversity, composition, and functional profile of the microbiota of toddlers that have undergone a long period of breastfeeding. Weaning during infancy is known to have a strong impact in the development of the gut microbiota, but our study shows that weaning also has an important effect when it occurs at a more advanced age, when children already intake large quantities of a variety of other foods. Cessation of breastfeeding favored the convergence of the microbiota of toddlers towards adult-like properties, but other factors must also have contributed to generate levels of diversity and stability similar to those of adults, as these were reached before most of the children in the study were weaned. Such factors might include increased maturity of the intestinal environment and immune system, as well as diet diversification. Moreover, although the microbiome reached an adult-like stability at around 2 years of age, important taxonomic and functional changes did occur both before and after adolescence. Therefore, adolescence can still be considered a transitional period, in which some taxa and functions have yet to attain the abundances observed in adults, so that the final stages of development occurring during this time may still be important for the establishment of a healthy adult microbiota. Furthermore, the specificities of the gut microbiota in adolescents should be taken into account when analyzing possible alterations due to disease or other factors in this age group.

## Data Availability

The datasets presented in this study can be found in online repositories. The names of the repository/repositories and accession number(s) can be found at: https://www.ebi.ac.uk/ena, these submission numbers by the Study Accession Number PRJEB101630.
