# Supplementary material for: Patterns of gut microbiome composition, function and dynamics in toddlers, adolescents and adults over a three-year period
Source: Front Microbiol. 2026 Mar 20;17:1768977. doi: 10.3389/fmicb.2026.1768977 (PMC13047174; doi:10.3389/fmicb.2026.1768977)
Supplement: Supplementary file 1 [file Data_Sheet_1.DOCX]

Supplementary Material

# Supplementary Figures and Tables

## Supplementary Figures


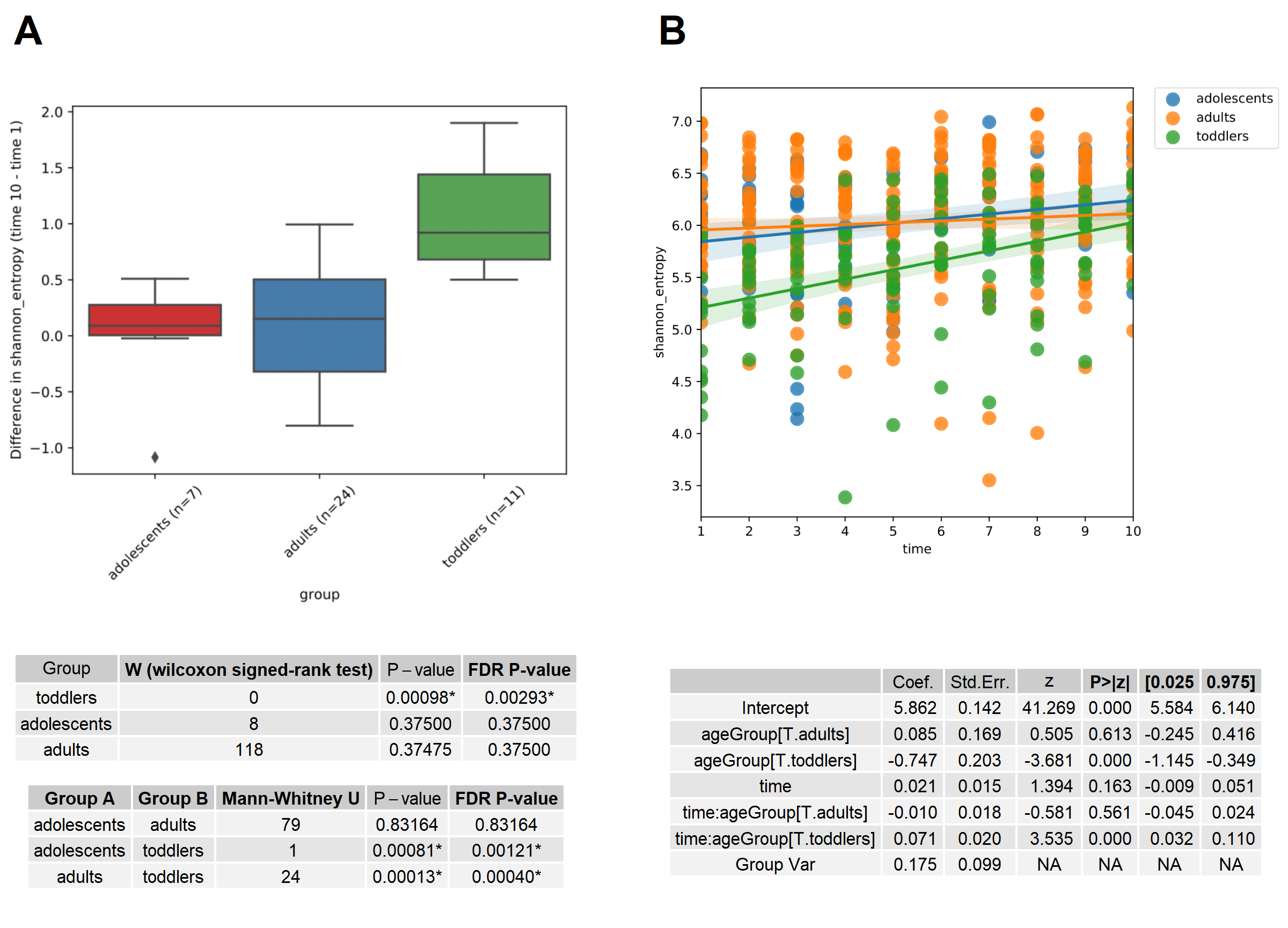


**Supplementary Figure 1.** **Temporal variability of microbial diversity.** Analysis of time series compositional data with QIIME. **(A)** Difference in alpha diversity values (Shannon index) between the first time point (T1) and the last one (T10) (intra-group Wilcoxon signed-rank test) and comparison of the T10-T1 difference between age groups (inter-group Mann-Whitney U test). **(B)** Linear Mixed Effects models to test the changes in alpha diversity (Shannon index) over time.

## Supplementary Tables

All Supplementary Tables are found in a single Excel File uploaded under the filename Supplementary_Tables_S1-S9.

**Table S1.** Metadata for volunteers and samples.

**Table S2.** 16S rRNA sequencing statistics per sample.

**Table S3.** Metagenome sequencing statistics per sample.

**Table S4.** p-values of diversity (Shannon index and Chao1 estimator) for pairwise comparisons between groups and between timepoints.

**Table S5.** Canonical Correspondence Analysis (CCA) and PERMANOVA (Adonis function) p-values.

**Table S6.** List of ASVs considered as discriminative of each age group by spls-da.

**Table S7.** List of TIGRFAMs considered as discriminative of each age group by spls-da.

**Table S8.** List of ASVs and TIGRFAM IDs associated to the *Circos* plot and their feature annotations.

**Table S9.** p-values of pairwise comparisons of the Jaccard index (fraction of shared features between consecutive samples of the same individual) for different time intervals within each age group and for the same time interval between groups, at ASV and TIGRFAM level.
